# Monosodium urate burden assessed with dual-energy computed tomography predicts the risk of flares in gout: a 12-month observational study: MSU burden and risk of gout flare

**DOI:** 10.1186/s13075-018-1714-9

**Published:** 2018-09-17

**Authors:** Tristan Pascart, Agathe Grandjean, Benoist Capon, Julie Legrand, Nasser Namane, Vincent Ducoulombier, Marguerite Motte, Marie Vandecandelaere, Hélène Luraschi, Catherine Godart, Eric Houvenagel, Laurène Norberciak, Jean-François Budzik

**Affiliations:** 1 Department of Rheumatology, Lille Catholic Hospitals, University of Lille, 59160 Lomme, France; 2 Department of Radiology, Lille Catholic Hospitals, University of Lille, 59160 Lomme, France; 3 Department of Medical Research, Biostatistics, Lille Catholic Hospitals, University of Lille, 59160 Lomme, France; 4 EA 4490, PMOI, Physiopathologie des Maladies Osseuses Inflammatoires, University of Lille, 59000 Lille, France; 5 Saint-Philibert Hospital, Rue du Grand But, 59160 Lomme, France

**Keywords:** Gout, Flares, Dual-energy computed tomography, Ultrasonography

## Abstract

**Background:**

Predicting the risk of flares in patients with gout is a challenge and the link between urate burden and the risk of gout flare is unclear. The objective of this study was to determine if the extent of monosodium urate (MSU) burden measured with dual-energy computed tomography (DECT) and ultrasonography (US) is predictive of the risk of gout flares.

**Methods:**

This prospective observational study recruited patients with gout to undergo MSU burden assessment with DECT (volume of deposits) and US (double contour sign) scans of the knees and feet. Patients attended follow-up visits at 3, 6 and 12 months. Patients having presented with at least one flare at 6 months were compared to those who did not flare. Odds ratios (ORs) (95% confidence interval) for the risk of flare were calculated.

**Results:**

Overall, 64/78 patients included attended at least one follow-up visit. In bivariate analysis, the number of joints with the double contour sign was not associated with the risk of flare (*p* = 0.67). Multivariate analysis retained a unique variable: DECT MSU volume of the feet. For each 1 cm^3^ increase in DECT MSU volume in foot deposits, the risk of flare increased 2.03-fold during the first 6 months after initial assessment (OR 2.03 (1.15–4.38)). The threshold volume best discriminating patients with and without flare was 0.81 cm^3^ (specificity 61%, sensitivity 77%).

**Conclusions:**

This is the first study showing that the extent of MSU burden measured with DECT but not US is predictive of the risk of flares.

**Electronic supplementary material:**

The online version of this article (10.1186/s13075-018-1714-9) contains supplementary material, which is available to authorized users.

## Background

Gout is a metabolic condition related to monosodium urate (MSU) crystal deposition in joints and soft tissue leading to NLRP inflammasome-guided recurrent arthritis [1]. Despite the permanent deposition of MSU crystals, flares of inflammatory response are only intercurrent and difficult to predict. Risk factors for gout flares are known or suspected but causality is poorly understood [2, 3].

Initiation and modifications of urate lowering therapy (ULT) are critical times for flares. Prophylaxis of flares with colchicine, non-steroidal anti-inflammatory drugs (NSAIDs) or even oral corticosteroids is recommended by all international guidelines during 6 months following ULT initiation [4–6]. These recommendations are based upon the observation of an increased rate of flares during the first 6 months of ULT in most randomized controlled trials (RCTs) involving ULTs versus placebo [7, 8]. Prophylaxis, especially with colchicine, has been proven effective to decrease the risk of flares [9–11]. It is generally accepted that the reduced serum urate (SU) [12] concentration induces mobilization of the deposited MSU burden, potentially exposing the crystals to the innate immune system [11, 13]. This explanation, however, remains hypothetical.

Ultrasonography (US) and dual-energy computed tomography (DECT) can provide an assessment of the MSU burden [14]. DECT uses two x-ray beams with two different energies allowing to distinguish between urate and calcium in soft tissues surrounding bone, with radiation exposure close to conventional computed tomography (CT) [15]. US, on the other hand, can identify intra-articular cartilage MSU deposition appearing as a double contour (DC) sign, which disappears during urate depletion [16]. The usefulness of US and DECT is now fully recognized for the diagnosis of gout [17]. The clinical relevance of observing and measuring the urate burden by any imaging technique however needs to be determined. Previous data suggest an association between urate burden and past flares [18] but to our knowledge no imaging feature has been associated with the risk of flares [19]. More generally, no data have so far demonstrated that imaging adds to the low cost clinical and biological assessments in the management of gout [19].

The objective of this study was to determine if the extent of urate burden measured with DECT and US predicts the risk of gout flares.

## Methods

### Patients

This prospective observational study included consecutive patients with a diagnosis of gout according to the American College of Rheumatology (ACR)/European League Against Rheumatism (EULAR) 2015 criteria [17]. They were recruited to undergo quantification of urate deposition of the knees and feet using US and DECT [14, 20] and were subsequently followed in outpatient visits. The study was approved by the institutional review board of the Lille Catholic Hospitals and all participants provided informed consent before inclusion into the study.

### Visits

Patients were seen for their follow up as decided by their physician. The initial (M0) visit was contemporary with the US and DECT scans. Demographic characteristics, disease history, treatments and biological data were then collected. Physicians were unaware of the results of the DECT scans results during follow up.

The three follow-up visits examined were those performed at months 3 (M3), 6 (M6) and 12 (M12) (± 1 month allowed). The number of flares since M0 and since the previous timepoint were recorded at each visit. Flare definition was based upon the patient’s own assessment according to previous experiences of flares and retrospectively validated by the investigator upon the description of the episode [21]. Data on type and dose of ongoing ULT and flare prophylaxis were collected. Contemporary SU levels were noted when available.

### DECT scans

All scans were performed using a single-source CT (Somatom Definition Edge; Siemens, Erlangen, Germany). Knees and feet were scanned axially in two separate acquisitions performed consecutively on the same day. All scans were performed with the same image protocol, with acquisition at 128 × 0.6 mm and pitch at 0.7. Two scans of each body region were acquired with tube potentials of 80 kV and 140 kV. Depending on the scanned body region, quality reference tube currents ranged between 62 and 260 mAs. Automated attenuation-based tube current modulation was used in all examinations.

Axial images with soft (B30f) and bone (B70f) convolution kernels were reconstructed with 1 mm slice thickness and 1 mm increment. Dedicated software (syngo.via VB10B, syngo Dual Energy Gout; Siemens) was used for DECT post-processing, following parameters described elsewhere [22]: HouNsfield unit (HU) threshold, 150; iodine ratio, 1.4; material definition ratio, 1.25; resolution, 4; air distance, 5; bone distance, 10. Two kinds of images were reconstructed for each body region. First, volume-rendered 3D images in which urate crystal deposits coded in green were reconstructed with a bone tissue convolution kernel (B70f). These images allowed a straightforward overview of MSU deposits. Second, multi-planar reformations associating images reconstructed with a soft tissue kernel (B30f) and colored images were reconstructed. The aspect of the final fusion images could be changed by modulating the relative percentages of the morphological and colored images from 0 to 100% with a slider. MSU deposits above 20 cm^3^ in patients assessed by DECT were considered extreme and these patients were excluded to avoid over-estimated weight in the OR computations of the risk of flares per unit of MSU volume.

### US scans

Examinations were performed by one of four trained musculoskeletal radiologists on an Applio 400 US machine (Toshiba Medical Systems, Tochigi, Japan). High-frequency probes were used: a 12 Mhz probe for the knee examination and an 18 MHz probe for the ankle and foot examination. The patellofemoral joints, talocrural joints and 1st metatarsophalangeal joints were examined by US for the DC sign as defined by Outcome Measures in Rheumatology (OMERACT) [23].

### Statistical analysis

All statistical analyses were performed using R version 3.4. Qualitative variables were described as numbers (%) of each response modality; quantitative variables were described as mean ± SD.

Two groups were defined according to the occurrence of flares between M0 and M6: the first group included patients having presented with no flare, the second those having presented with at least one. Group-variable bivariate analysis was applied to search for factors predictive of flaring. Variables assessed were DECT MSU volume in the feet at M0, DECT MSU volume in the knees at M0, SU time course from M0 to M6, use of prophylaxis at M6, number of joints with the DC at M0, use of ULT at M0 and number of flares per year at M0. The chi-square test or Fisher’s exact test, as appropriate, was used for comparative analysis of proportions. Continuous variables were compared using Student’s *t* test for normal data, or the Mann-Whitney-Wilcoxon test. Multivariate analysis was then applied to search for factors affecting group assignment: a binary logistic regression model explaining the group and integrating all these data was constructed (a “complete” model). The small sample size required a variable selection procedure. The step by step, backward method, based on the Akaike criterion, was chosen to obtain a “reduced” model. Validation and reduced model performance were assessed by the ROC area under the curve. As they were satisfactory, the odds ratios (ORs) of explanatory variables selected by the automatic selection and their 95% confidence intervals are presented. The cutoff best discriminating between the groups was deduced from the ROC curve. Sensitivity and specificity were calculated according to this threshold. As a single variable was selected by the automatic selection procedure, a further OR analysis was performed of the ORs of presenting with at least one flare for each period between time points (namely M0–M3, M0–M6, M0–M12 and M6–12) based upon the entire sample of patients attending the analyzed visits with the available number of flares over the considered period of time. These ORs were obtained using a simple binary logistic regression model integrating this unique variable. The significance level was set at 5%.

## Results

### Patients

Overall, 78 patients were included in the study and had an initial assessment of urate burden with DECT and US scans. Their characteristics and those of the groups of patients who experienced at least one flare or not during the first 6 months of follow up are described in Table 1.

**Table 1:** Population characteristics and characteristics of patients flaring and not flaring between month 0 (M0) and month 6 (M6) (significance of the *p* value set at 5%)

Characteristics	Population (*n* = 78)	Patients without flare between M0 and M6 (*n* = 33)	Patients with at least 1 flare between M0 and M6 (*n* = 19)	*p* value
Demographics				
Age (years)	64.8 ± 13.9	67.4 ± 12.3	67.7 ± 13.8	0.95
Male	68 (87.2%)	26 (78.8%)	17 (89.5%)	0.46
Body mass index (kg/m^2^)	28.9 ± 4.3	29.1 ± 4.5	28.7 ± 4	0.62
Current smoking	10 (12.8%)	2 (6.1%)	4 (21.1%)	0.18
Excessive alcohol consumption (*n*)	38 (48.7%)	16 (48.5%)	8 (42.1%)	0.88
Creatinine clearance (mL/min)	76.8 ± 28.0	70.3 ± 28.2	77.5 ± 32.8	0.51
Comorbidities				
High blood pressure	43 (55.1%)	22 (66.7%)	5 (26.3%)	**0.012**
Coronary heart disease	12 (15.4%)	2 (6.1%)	4 (21.1%)	0.18
Peripheral arterial disease	3 (3.8%)	0 (0%)	0 (0%)	1
Chronic heart disease	18 (23.1%)	8 (24.2%)	2 (10.5%)	0.29
Stroke	8 (10.3%)	5 (15.2%)	0 (0%)	0.15
Dyslipidemia	37 (47.4%)	18 (54.5%)	8 (42.1%)	0.56
Diabetes mellitus	20 (25.6%)	12 (36.4%)	3 (15.8%)	0.21
Obstructive sleep apnea	11 (14.1%)	6 (18.2%)	2 (10.5%)	0.69
Psoriasis	5 (6.4%)	3 (9.1%)	1 (5.3%)	1
Ongoing diuretics	19 (24.4%)	8 (24.2%)	4 (21.1%)	1
Disease characteristics				
Gout duration (years)	11.8 ± 11.9	9.6 ± 11.2	15.8 ± 13.4	0.061
Renal stones	13 (16.7%)	6 (18.2%)	3 (15.8%)	1
Family history of gout	18 (23.1%)	6 (18.2%)	6 (31.6%)	0.32
Declared number of flares over the past year	4.1 ± 5.9	3.2 ± 4.4	4.6 ± 5.8	0.15
Baseline ongoing flare prophylaxis	25 (32.1%)	12 (36.4%)	7 (36.8%)	1
Baseline serum urate (mg/dL)	7.43 ± 2.33	6.93 ± 2.14	7.85 ± 2.12	0.096
Ongoing urate lowering therapy	36 (46.2%)	16 (48.5%)	9 (47.4%)	1
Allopurinol	*18 (50%)*	8 (50%)	5 (55.6%)	0.38
Febuxostat	*16 (44.4%)*	8 (50%)	3 (33.3%)	
Probenecid	*1 (2.8%)*	0 (0%)	1 (11.1%)	
Benzbromarone	*1 (2.8%)*	0 (0%)	0 (0%)	
Subcutaneous tophi	28 (35.9%)	7 (21.2%)	9 (47.4%)	0.098
US tophi	49 (62.8%)	19 (57.6%)	13 (68.4%)	0.63
Number of joints with the US double contour sign (/6)	2.4 ± 1.3	2.2 ± 1	2.8 ± 1.4	0.3
At least one US double contour sign	75 (96.2%)	32 (97%)	19 (100%)	1
DECT MSU volume knees (cm^3^)	6.3 ± 28.1	0.6 ± 1.3	1.7 ± 3.4	0.11
DECT MSU volume feet (cm^3^)	5.4 ± 16.7	0.9 ± 1.3	2.4 ± 2.1	**0.0064**

*US* ultrasonography, *DECT* dual-energy computed tomography, *MSU* monosodium urate

Significance of the *p* value was set at 5% are in bold

Of these patients, 14 were lost to follow up, 2 were excluded because of extreme volumes of MSU deposits (volumes > 75 cm^3^) and the remaining 62 patients attended at least one of the three follow-up visits. Data recorded at each visit are presented in Table 2.

**Table 2:** Details of the clinical, biological and treatment data collected during each follow-up visit

	Month 0	Month 3	Month 6	Month 12
Patients attending the visit	78 (100%)	42 (65.6%)	54 (84.4%)	38 (59.4%)
Serum urate (mg/dL)	7.43 ± 2.33	6.57 ± 1.9	5.59 ± 1.56	5.63 ± 1.81
Ongoing ULT	36 (46.2%)	33 (78.5%)	47 (87.1%)	34 (89.4%)
Ongoing flare prophylaxis	25 (32.1%)	20 (47.6%)	27 (50%)	16 (42.1%)
Number of flares since M0	N/A	0.6 ± 1.3	0.7 ± 1.4	1.5 ± 3.3
Patients with at least 1 flare since M0	N/A	15 (35.7%)	19 (35.2%)	16 (42.1%)
Number of flares since previous visit	N/A	0.6 ± 1.3	0.3 ± 0.7	0.7 ± 1.6
Patients with at least 1 flare since previous visit	N/A	15 (35.7%)	9 (17%)	12 (31.6%)

*M0* month 0, *ULT* urate lowering therapy

At M6, 27/54 patients (50%) were receiving flare prophylaxis. Of these 27 patients, 10 (38.5%) were receiving 0.5 mg colchicine daily, 12 (46.2%) 1 mg colchicine daily,1 full dose NSAIDs (3.8%), 1 oral corticosteroids (3.8%) and the last 2 patients were treated with anakinra (7.7%). A total of 47/54 patients were receiving ULT, among whom 27 participants (57.4%) were treated with allopurinol, 17 with febuxostat (36.2%), 2 with benzbromarone (4.3%) and 1 with probenecid (2.1%). At M6, 19 patients (35.2%) had presented with at least one flare since baseline.

### Risk factors for flares

Overall, 36 patients without any missing data were seen at M6 and included in the multivariate analysis. The selected potential risk factors for flares during the 6 months following the initial assessment were tested for and presented in Table 3.

**Table 3:** Comparison in bivariate analysis of potential risk factors influencing the risk of presenting with at least one flare during the first 6 months of follow up of patients with all data available

	No flare between M0 and M6	At least 1 flare between M0 and M6	*p* value
Number of patients	23	13	/
DECT MSU volume knees (cm^3^)	0.6 ± 1.2	1.1 ± 1.6	0.24
DECT MSU volume feet (cm^3^)	0.9 ± 0.8	2.1 ± 1.9	0.05
Serum urate change between M0 and M6 (mg/dL)	1.51 ± 2.03	1.50 ± 2.48	0.88
Number of joints with the US double contour sign at M0 (/6)	2.5 ± 0.9	2.9 ± 1.5	0.67
Declared number of flares over the past year	3 ± 4.4	4.2 ± 6.2	0.41
Ongoing flare prophylaxis at M6	11 (47.8%)	6 (46.2%)	1
Ongoing urate lowering therapy at M0	13 (56.5%)	6 (46.2%)	0.8

*M0* month 0, *M6* month 6, *US* ultrasonography, *DECT* dual-energy computed tomography, *MSU*, monosodium urate

The volume of MSU deposition of the feet measured with DECT was the only factor significantly associated with the risk of flares in the bivariate analysis (*p* = 0.05). Moreover, this DECT volume was the only variable retained by the automatic selection procedure in the multivariate analysis (reduced model). The box plot of MSU volumes of the feet in the group of patients having presented with at least one flare between M0 and M6 and the one of those who did not flare are shown in Additional file 1: Figure S1.

**Fig. 1:**
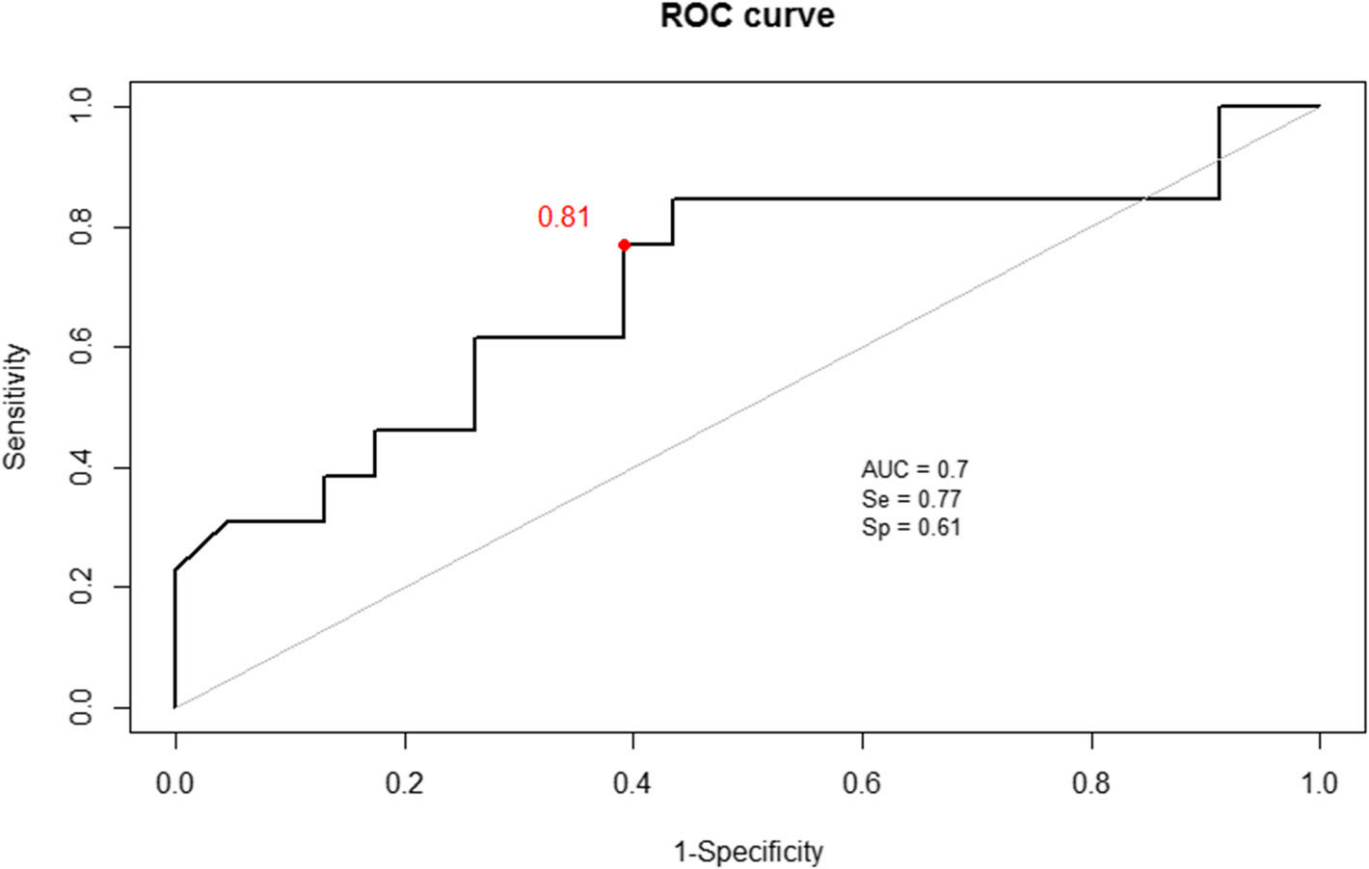
ROC curve explaining the risk of presenting with at least one flare during the first 6 months of follow up, with the monosodium urate volume deposited in the feet measured with dual-energy computed tomography. The red dot indicates the volume providing the best discrimination between the group of patients presenting with at least one flare and those without flare. The associated predictive values for this volume are shown. AUC, area under the curve; Sp, specificity; Se, sensitivity

**Fig. 2:**
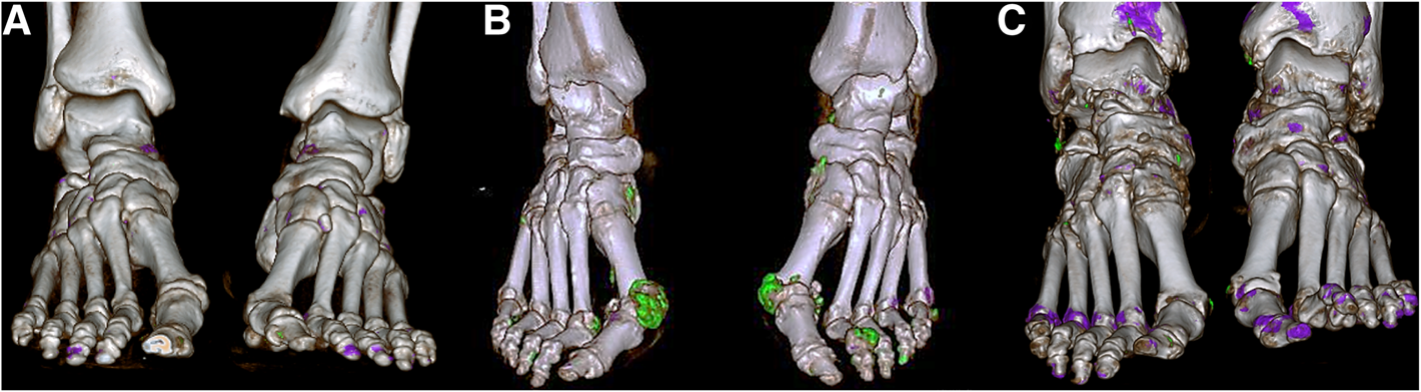
Dual-energy computed tomography imaging of monosodium urate crystal deposition in the feet. Small (volume 0.17 cm^3^) (**a**), large (volume 5.29 cm^3^) (**b**) and cutoff (**c**) soft tissue volume of deposits in patients with flare and without flare (volume 0.81 cm^3^)

The OR obtained in the reduced model was 2.03 with a 95% confidence interval of 1.15–4.38. The risk of developing a flare during the 6 months following initial assessment increased 2.03-fold for each 1 cm^3^ increase in MSU volume in deposits in the feet. The threshold volume best discriminating patients with and without flare during the 6 months following initial assessment was 0.81 cm^3^, with 61% specificity and 77% sensitivity (Figs. 1 and 2).

### Risk of flares between time points according to MSU volume

Having identified the DECT MSU volume of deposits in the feet as the unique significant factor predictive of flares, the ORs of having at least one flare were calculated for periods between time points. All patients with recorded data on the number of flares during each period of time were included in the analysis.

The ORs for risk of flaring between time points are presented in Table 4. The difference in the risk of flaring - associated with increased deposit volume in the feet determined by DECT at M0 - was significant between M0 and M6 and between M0 and M12, but not between M0 and M3 nor between M6 and M12. It is noteworthy that considering all 52 patients who attended the M0 and M6 visits (instead of the 36 patients with all data available who could be included in the multivariate analysis), the OR of flaring between M0 and M6 associated with increased MSU deposit volume was 1.69 (1.17–2.77).

**Table 4:** Odds ratios for the risk of presenting with at least one flare during follow up between each time point

Time period	Group	Number	M0 DECT MSU volume feet (cm^3^)	OR	(95% CI for OR)
M0–M6	0 flares	33	0.9 ± 1.3	**1.69**	**(1.17–2.77)**
	≥ 1 flare	19	2.4 ± 2.1		
M6–M12	0 flares	25	1.4 ± 1.7	1.13	(0.74–1.71)
	≥ 1 flare	12	1.8 ± 1.7		

*M* month, *DECT* dual-energy computed tomography, *MSU* monosodium urate, *OR* odds ratio, *CI* confidence interval

Significance of the OR with 95% confidence interval not including 1 are in bold

## Discussion

This study demonstrates that the extent of the MSU burden measured with DECT but not US predicts the risk of flares. Interestingly, patients with dramatically large volumes of MSU deposition are not necessarily at risk of flare. This is the first study showing the usefulness of DECT for the management of patients with gout, beyond diagnosis. These results also provide proof of concept that urate load is associated with the risk of flares.

With an OR of 2.03, the MSU burden in the feet assessed with DECT is the strongest determinant of subsequent gout flares identified so far. Some comorbidities, namely hypertension, renal disease and coronary heart disease, have been identified in a population-based study as having hazard ratios of 1.1–1.3 of incident gout flares [3]. Of note, patients with high blood pressure (HBP) were less at risk of flare in our study (Table 1). SU change from baseline has been shown to have an impact on the risk of flares in the febuxostat trials, but the OR was close to 1. This is consistent with our results as changes in SU levels between M0 and M6 were similar between participants experiencing and not experiencing flares. Rapidly obtaining SU levels below the dissolution threshold of MSU was associated with an OR of 1.42 of incident flares in the febuxostat trials and could be related to the mobilization of the MSU burden [13]. Prevalence of the US DC sign provides a different assessment of MSU burden than the one provided with DECT [14] and does not seem to predict the risk of flares. Flare prophylaxis is known to imperfectly prevent gout flares during ULT initiation [11]. The variety of situations included in our study, both in terms of ULT (absence, dose adaptation, initiation) and types of prophylaxis, would probably explain why it did not exhibit a preventive effect.

When the question of introducing or pursuing flare prophylaxis arises, DECT could be a decisive partner in the decision-making process. So far, international societies recommend flare prophylaxis during 6 months or until tophus resolution [4–6]. Becker et al. had shown from the febuxostat trials that the presence of subcutaneous tophi was one of the determinants of flare incidence during follow up [13]. Physical examination of tophi can be sufficient to show the presence of a significant MSU burden requiring flare prophylaxis. However, only approximatively 15–30% of patients present with subcutaneous tophi [24, 25]. Our study shows that DECT can provide an assessment of the risk of flares whether subcutaneous tophi are present or not. DECT scanning could be decisive when considering the interruption of flare prophylaxis after 6 months or when tophi are no longer clinically detectable.

MSU burden could be the missing link between ULT and flare reduction. So far, SU has been the primary endpoint in the majority of ULT trials, as observational data consistently show that eventually flare reduction correlates with reduction in SU [26]. In its latest highly debated guidelines, the American College of Physicians has challenged the relevance of targeting SU rather than flares themselves, considering that the evidence linking SU and flares is insufficient in RCTs [26–29]. For instance, the double blind RCTs for the development of febuxostat failed to show flare reduction with febuxostat [30, 31]. Similarly, another RCT of febuxostat versus placebo in early gout showed no reduction after 6 months of treatment [8]. Given that it has already been shown that ULT in its most potent form (pegloticase) decreases the MSU burden assessed with DECT and that our study demonstrates that MSU volumes measured with DECT predict gout flares, DECT could be used as a clinically relevant outcome measure [32]. Depending on the kinetics of MSU depletion with conventional ULTs (hypoxanthine oxidase inhibitors and uricosurics), DECT MSU volumes could be considered as a surrogate marker for the risk of flares potentially more efficiently than SU.

We acknowledge that the study design had some limitations, imposing caution while interpreting the results. First, although an effect of volume of the MSU burden measured by DECT on the risk of flares was detected despite the small sample size, the rather small number of patients and the missing data may have underestimated other factors with a smaller effect. Particularly, the study did not detect the expected significant effect of the change in SU levels on the risk of flares [33]. However, the SU change between patients with and without flare was very similar in our study, suggesting that the link between change in SU level itself and the risk of flares may not be as direct as previously hypothesized. For instance, the high risk of flares during pegloticase therapy (and to a lesser extent during less potent ULTs) could be more the reflection of rapid depletion in MSU burden than SU change itself [32, 34, 35]. Second, the study included a panel of treatment initiators, patients already treated needing reinforced therapy and patients remaining under stable ULT treatment, which does not allow us to determine if flare prediction by DECT could be applicable at all stages in gout management. Future studies should assess the relationships operating in each of these subpopulations. Third, the specificity and sensitivity of the 0.81 cm^3^ cutoff for MSU deposits in the feet above which there is a significant risk of flaring were probably over-estimated, given the fact that they were calculated based upon the sample that was also used to build the model. Specificity and sensitivity testing in another population is needed. Fourth, 14 patients were lost to follow up which is expected in a population with gout who are known to have compliance issues. In addition, patients did not attend all visits, which may have led to a selection bias towards patients who experienced flare. Fifth, definition of gout flares in this study relied on patient’s self-assessment and retrospective confirmation by the physician. Although gout flares are marking painful experiences, the recently published definition of flares was not applied as such and the recollection of having experienced a flare may have been wrong for some patients [21, 36]. Sixth, the accuracy of MSU volume measurement with DECT is still debated, but measurements were standardized and known artifacts removed [37–39]. Finally, patients were assessed at each visit but there was no assessment of treatment adherence (prophylaxis and ULT) between visits that could potentially have an unmeasured impact on the risk of flares. The same can be said for known triggers for gout attacks such as alcohol intake or intercurrent infection. It can only be assumed that patient behavior was similar between groups.

## Conclusions

We believe this study is a step forward in our ability to tailor gout management to individual patients’ initial profiles. Particularly, special caution should be given in the prevention of flares in patients with initial high urate burden.

## Additional file

Additional file 1: **Figure S1.** Box plot of the initial volume of monosodium urate deposits in the feet measured with dual-energy computed tomography for the group of patients presenting with at least one flare and those without flare during the first 6 months of follow up. (TIF 792 kb)

## Abbreviations

ACR: American College of Rheumatology
CT: Computed tomography
DC: Double contour
DECT: Dual-energy computed tomography
EULAR: European League Against Rheumatism
M: Month
MSU: Monosodium urate
NSAID: Non-steroidal anti-inflammatory drug
OMERACT: Outcome Measures in Rheumatology
OR: Odds ratio
RCT: Randomized controlled trial
ROC: Receiver operator characteristic
SU: Serum urate
ULT: Urate lowering therapy
US: Ultrasonography
