# Supplementary figures and images for: Monosodium urate burden assessed with dual-energy computed tomography predicts the risk of flares in gout: a 12-month observational study: MSU burden and risk of gout flare
Source: Arthritis Res Ther. 2018 Sep 17;20:210. doi: 10.1186/s13075-018-1714-9 (PMC6142357; doi:10.1186/s13075-018-1714-9)

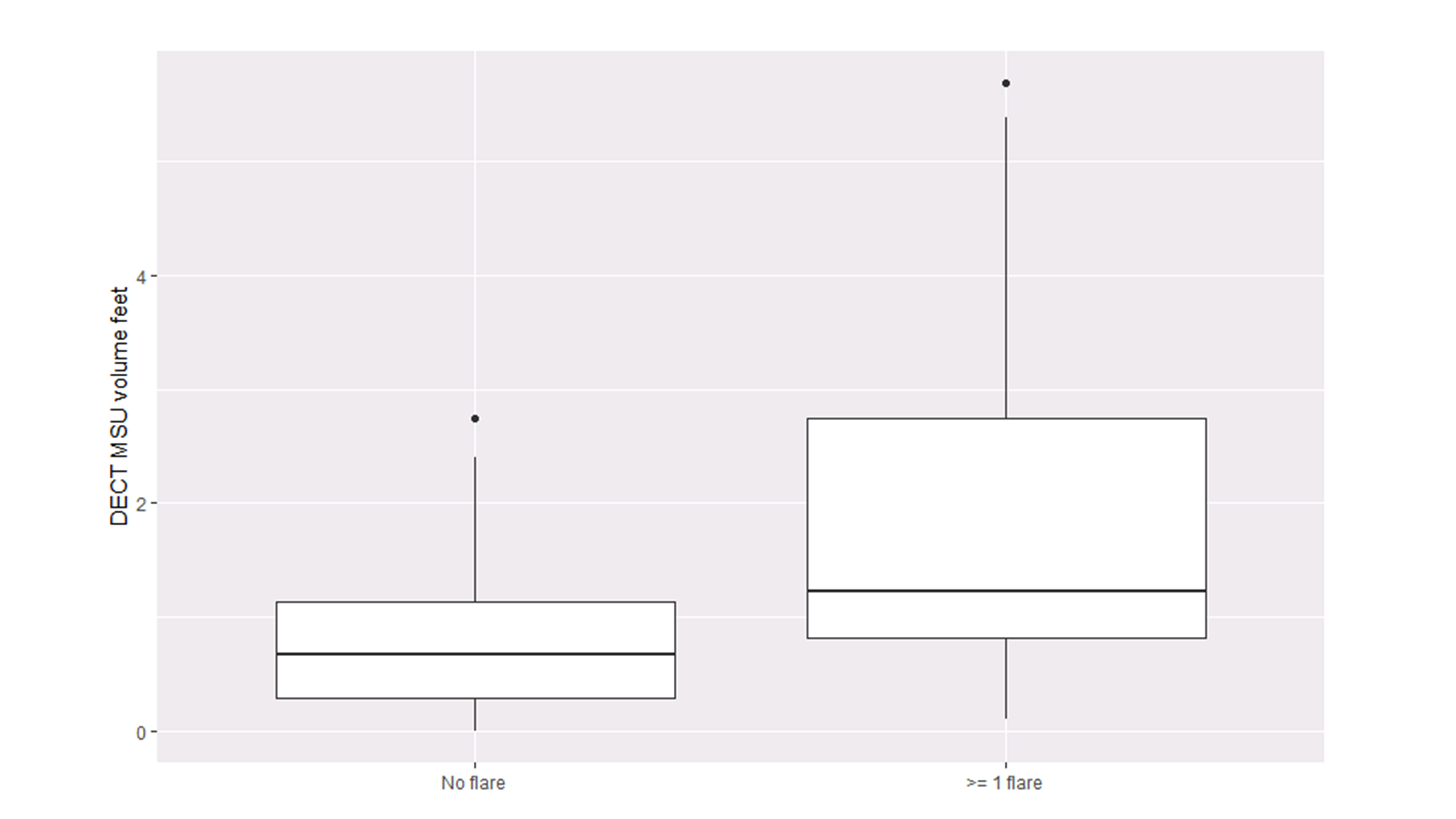

Supplement: Supplementary file 1 — Figure S1. Box plot of the initial volume of monosodium urate deposits in the feet measured with dual-energy computed tomography for the group of patients presenting with at least one flare and those without flare during the first 6 months of follow up. (TIF 792 kb) [file 13075_2018_1714_MOESM1_ESM.tif]
